# Supplementary material for: Divergent evolutionary strategies pre-empt tissue collision in gastrulation
Source: Nature. 2025 Sep 3;646(8085):637–46. doi: 10.1038/s41586-025-09447-4 (PMC12527943; doi:10.1038/s41586-025-09447-4)
Supplement: Supplementary file 1 — Supplementary notes, bibliography and methods. [file 41586_2025_9447_MOESM1_ESM.pdf]

nature portfolio

https://doi.org/10.1038/s41586-025-09447-4

Supplementary information

Divergent evolutionary strategies pre-empt tissue collision in gastrulation

In the format provided by the authors and unedited

Divergent evolutionary strategies preempt tissue collision in gastrulation

Bipasha Dey[1,4], Verena Kaul[2,3,4], Girish Kale[2,3,4], Maily Scorcelletti[2], Michiko Takeda[1], Yu-Chiun Wang[1,5], Steffen Lemke[2,3,5]

[1]RIKEN Center for Biosystems Dynamics Research, Kobe, Japan
[2]Centre for Organismal Studies Heidelberg, Heidelberg University, Heidelberg, Germany
[3]Institute of Biology, Department of Zoology, University of Hohenheim, Stuttgart, Germany
[4]These authors contributed equally.
[5]Correspondence: Y.-C.W. (yu-chiun.wang@riken.jp) and S.L. (steffen.lemke@uni-hohenheim.de).

Table of Contents

Supplementary Notes 3

Supplementary Bibliography 6

Supplementary Methods 11

Supplementary Notes

1. The expression patterns of *Cri-btd* and *Cri-eve*

In the blastoderm, *Cri-btd* is expressed in two separate head domains and *Cri-eve* in six stripes (Fig. 1k, l). A seventh stripe of *Cri-eve* posterior to the first six stripes appears during gastrulation, similar to previously described pattern for the *eve* ortholog in the moth midge *C. albipunctata* (Fig. 1a, Psychodidae; Rohr *et al.* 1999 Dev. Genes Evol. 209, 145–154).

2. On the efficacy and variability of phenotypic effects associated with *btd* dsRNA injection

The efficacy of all *btd* dsRNA preps were verified based on phenotypes. Specifically, the blocking of CF formation and the resultant occurrence of head-trunk buckling were comparable to those observed in the btd^{AX} homozygous embryos. When combined with *stg* mutants or InscOE, we consistently observed two classes of phenotypes (Fig. 3d, e and Fig. 6e-g) despite raising the concentrations of *btd* dsRNA by ~10 fold. Technical difficulties prevented us from executing these experiments using the btd^{AX} mutant as our attempt of generating a stable stock of *btd stg* double mutants that also contains both a membrane imaging marker and fluorescent marked (hb0.7-Venus-NLS) balancers for genotyping was unsuccessful. Thus, we were not able to fully resolve the differences between our data and those of the companion study (Vellutini *et al.* 2025) regarding the phenotypes of *btd stg* double loss of function.

3. On assessing embryonic lethality following optogenetic ablation of the CF

To approximate the fitness of embryos that fail to form the CF, we initially planned to use the experimental design (3) described in the Optogenetics section in Methods to eliminate the CF, followed by scoring the embryonic hatching rate. The opto-DNRho1 embryos used for these experiments indeed hatched into larvae if left on the embryo collection agar plate and kept in the dark without any further handling or perturbation. In contrast, however, embryos that underwent the standard embryo mounting procedure – dechorionated, affixed to a glass bottom dish with the heptane glue, and immersed under a mixture of Halocarbon oil 700 and 27 in the ratio 3:1 – and kept in a moisturized chamber at 22°C in the dark for 24-48 hours without imaging or any photostimulation, showed a low hatching rate (36%; 6 trials; 5 embryos/trial), contrasting with wild-type (Oregon R) embryos that scored a 91% hatching rate (4 trials; 10 embryos/trial). These data suggest that the currently available tool for

optogenetic inhibition of the CF, i.e., the flies that carry the opto-DNRho1 system, has high background lethality even without photostimulation, thus unsuitable for the evaluation of embryonic lethality following CF ablation.

4. Characterization and designation of *C. riparius* MDs

We characterized head mitosis in the *C. riparius* embryo to classify and provide MD nomenclature in accordance with the *D. melanogaster* naming convention, where the domain number is given chronologically. We designated the anterior-most region of the head as MD1, where the first dividing cells are observed, and divisions in a large lateral domain posterior to MD1 as MD2 (Fig. 5b). Although we observed out-of-plane divisions in MD1 (data not shown), high curvature in the anterior-most region makes it challenging to systematically analyze the division plane. We thus focused on MD2. MD2 cells begin rounding up for mitosis ~7.5 min after gastrulation starts, with the first telophase at ~13 min. Thus, head mitosis occurs soon after gastrulation onset and temporally overlaps with trunk expansion, comparable to *D. melanogaster* (Extended Data Fig. 9a).

5. Functional role of Insc in out-of-plane division

The mitotic spindle anchoring protein Insc is necessary and can be sufficient for out-of-plane division in the *Drosophila* embryonic epithelia. Widespread expression of *insc* in the *C. riparius* blastoderm (Extended Data Fig. 9d) and high percentage of out-of-plane division in the head MDs suggest a functional link between the two. Our attempts in testing this – via RNAi knockdown of *insc* – were unsuccessful in that we did not observe reorientation of the division plane from out-of-plane to in-plane in *C. riparius*. These data suggest Insc is just one of several redundant components controlling division orientation. For example, cortical stiffness of the mitotic cells in the *C. riparius* head may not be sufficiently high to resist compressive stress in the tissue, such that the default orientation of the spindle is out-of-plane, even in the absence of Insc. Alternatively, other dedicated, redundant spindle anchorage cues may exist. Our data also suggest that the evolutionary transition to in-plane division would require more than a mere restriction of *insc* expression. The presence of both out- and in-plane dividers within *C. riparius* head MDs despite broad expression of *insc* suggests potential volatility of division orientation in the Dipteran stem group. It would be of interest to quantitatively assess the variability and volatility of division orientation in the embryonic head in non-cyclorrhaphan flies.

6. Model: divergent evolutionary strategies preempt tissue collision in gastrulation

In Fig. 6h, we depict our model concerning how divergent evolutionary strategies preempt tissue collision in gastrulation. In cyclorrhaphan flies, tissue collision at the head-trunk boundary is preempted by out-of-plane tissue deformation via the CF. In non-cyclorrhaphan flies, in contrast, collision is prevented through altered division orientation in the head. Specifically, in the first phase, a large number of cells in the major mitotic domain in the head (MD2) divide out-of-plane (MD2o, purple), resulting in reduced expansion as compared to the in-plane dividing cells (MD2i, orange). Furthermore, in the second phase, once divisions are completed in MD2o, the tissue acts as a mechanical sink, allowing the subsequent expansion of MD2i into some of the space previously occupied by MD2o. Thus, even though trunk expansion driven by GBE is evolutionarily conserved, no buckling resulting from mechanical instabilities is observed despite the absence of preemptive out-of-plane deformation.

Supplementary Bibliography

This Supplementary Bibliography provides the full reference list for the literature survey reported in Extended Data Fig. 1.

Abbassy, Magda M., Nadia Helmy, Mostafa Osman, Stanton E. Cope, and Steven M. Presley. 1995. “Embryogenesis of the sand fly *Phlebotomus papatasi* (Diptera: Psychodidae): Cell cleavage, blastoderm formation, and gastrulation.” *Annals of the Entomological Society of America* 88 (6): 809–14. https://doi.org/10.1093/aesa/88.6.809.

Ajidagba, Peace, C. W. Pitts, and D. E. Bay. 1983. “Early embryogenesis in the stable fly (Diptera: Muscidae).” *Annals of the Entomological Society of America* 76 (4): 616–23. https://doi.org/10.1093/aesa/76.4.616.

Anderson, D. T. 1962. “ The embryology of *Dacus tryoni* (Frogg.) [Diptera, Trypetidae (= Tephritidae)], the Queensland fruit-fly .” *Development* 10 (3): 248–92. https://doi.org/10.1242/dev.10.3.248.

Blechert, O., D. Douglas, and S. Baumgartner. 2011. “Conserved function of the *Krüppel* gap gene in the Blowfly *Lucilia sericata*, despite anterior shift of expression.” *Insect Molecular Biology* 20 (2): 257–65. https://doi.org/10.1111/j.1365-2583.2010.01063.x.

Bois, Anne Marie Du. 1932. “A contribution to the embryology of Sciara (Diptera).” *Journal of Morphology* 54 (1): 161–95. https://doi.org/10.1002/jmor.1050540105.

Bownes, Mary. 1975. “A photographic study of development in the living embryo of *Drosophila melanogaster.*” *Development* 33 (3): 789–801. https://doi.org/10.1242/dev.33.3.789.

Butt, F. H. 1934. “Embryology of Sciara (Sciaridae: Diptera).” *Annals of the Entomological Society of America* 27 (4): 565–79. https://doi.org/10.1093/aesa/27.4.565.

Cantwell, George E., Anthony J. Nappi, and John George Stoffolano. 1976. “Embryonic and postembryonic development of the house fly (*Musca domestica* L.).” *U.S.Dep.Agric.Tech.Bull*, 1–69.

Caroti, Francesca, Silvia Urbansky, Maike Wosch, and Steffen Lemke. 2015. “Germ line transformation and in vivo labeling of nuclei in Diptera: Report on *Megaselia abdita* (Phoridae) and *Chironomus riparius* (Chironomidae).” *Development Genes and Evolution* 225 (3): 179–86. https://doi.org/10.1007/s00427-015-0504-5.

Carvalho, J.C, C.E Vanario-Alonso, T.M.C Silva, and E. Abdelhay. 1999. “Specialized features of *Rhynchosciara americana* embryogenesis.” *International Journal of Insect Morphology and Embryology* 28 (4): 309–19. https://doi.org/10.1016/S0020-7322(99)00040-9.

Davis, Catherine W.C., Johanna Krause, and Gerhard Krause. 1968. “Morphogenetic movements and segmentation of posterior egg fragments in vitro - *Calliphora erythrocephala* Meig., Diptera.” *Wilhelm Roux' Archiv für Entwicklungsmechanik der Organismen* 161 (3): 209–40. https://doi.org/10.1007/BF00573803.

Eritano, Anthony S., Claire L. Bromley, Antonio Bolea Albero, Lucas Schütz, Fu-Lai Wen, Michiko Takeda, Takashi Fukaya, et al. 2020. “Tissue-scale mechanical coupling reduces morphogenetic noise to ensure precision during epithelial folding.” *Developmental Cell* 53 (2): 212-228.e12. https://doi.org/10.1016/j.devcel.2020.02.012.

Gambrell, F. L., and Lydia A. Jahn. 1933. “The Embryology of the black fly, *Simulium pictipes* Hagen.” *Annals of the Entomological Society of America* 26 (4): 641–71. https://doi.org/10.1093/aesa/26.4.641.

García-Solache, Mónica, Johannes Jaeger, and Michael Akam. 2010. “A systematic analysis of the gap gene system in the moth midge *Clogmia albipunctata.*” *Developmental Biology* 344 (1): 306–18. https://doi.org/10.1016/j.ydbio.2010.04.019.

Genç, Hanife. 2014. “Embryonic development of the olive fruit fly, *Bactrocera oleae* Rossi (Diptera: Tephritidae), in vivo.” *Turkish Journal of Zoology* 38 (5): 598–602. https://doi.org/10.3906/zoo-1305-19.

Goltsev, Yury, William Hsiong, Gregory Lanzaro, and Mike Levine. 2004. “Different combinations of gap repressors for common stripes in *Anopheles* and *Drosophila* embryos.” *Developmental Biology* 275 (2): 435–46. https://doi.org/10.1016/j.ydbio.2004.08.021.

Havelka, Jan, Vladimír Jr. Landa, and Vladimír Landa. 2007. “Embryogenesis of *Aphidoletes aphidimyza* (Diptera: Cecidomyiidae): Morphological markers for staging of living embryos.” *European Journal of Entomology* 104 (1): 81–87. https://doi.org/10.14411/eje.2007.013.

Idris, Bahaa Eldien M. 1960. “Die Entwicklung im normalen Ei von *Culex pipiens* L. (Diptera).” *Zeitschrift für Morphologie und Ökologie der Tiere* 49 (4): 387–429. https://www.jstor.org/stable/43262061.

Jiménez-Guri, Eva, Karl R. Wotton, Brenda Gavilán, and Johannes Jaeger. 2014. “A staging scheme for the development of the moth midge *Clogmia albipunctata*.” Edited by Peter K. Dearden. *PLoS ONE* 9 (1): e84422. https://doi.org/10.1371/journal.pone.0084422.

Juhn, J., O. Marinotti, E. Calvo, and A. A. James. 2008. “Gene structure and expression of *nanos* (*nos*) and *oskar* (*osk*) orthologues of the vector mosquito, *Culex quinquefasciatus*.” *Insect Molecular Biology* 17 (5): 545–52. https://doi.org/10.1111/j.1365-2583.2008.00823.x.

Kaiser, Johannes, and Dirk F. Went. 1987. “Early embryonic development of the Dipteran insect *Heteropeza pygmaea* in the presence of cytoskeleton-affecting drugs.” *Roux’s Archives of Developmental Biology* 196 (6): 356–66. https://doi.org/10.1007/BF00375772.

Klomp, Jeff, Derek Athy, Chun Wai Kwan, Natasha I. Bloch, Thomas Sandmann, Steffen Lemke, and Urs Schmidt-Ott. 2015. “A cysteine-clamp gene drives embryo polarity in the midge *Chironomus*.” *Science* 348 (6238): 1040–42. https://doi.org/10.1126/science.aaa7105.

Kuntz, Steven G., and Michael B. Eisen. 2014. “Drosophila embryogenesis scales uniformly across temperature in developmentally diverse species.” *PLoS Genetics* 10 (4): e1004293. https://doi.org/10.1371/journal.pgen.1004293.

Lemke, Steffen, Stephanie E. Busch, Dionysios A. Antonopoulos, Folker Meyer, Marc H. Domanus, and Urs Schmidt-Ott. 2010. “Maternal activation of gap genes in the hover fly *Episyrphus*.” *Development* 137 (15): 2604–2604. https://doi.org/10.1242/dev.055558.

Lemke, Steffen, and Urs Schmidt-Ott. 2009. “Evidence for a Composite Anterior Determinant in the Hover Fly Episyrphus Balteatus (Syrphidae), a Cyclorrhaphan Fly with an Anterodorsal Serosa Anlage.” *Development* 136 (1): 117–27. https://doi.org/10.1242/dev.030270.

Martín-Vega, Daniel, and Martin J. R. Hall. 2016. “Estimating the age of *Calliphora vicina* eggs (Diptera: Calliphoridae): Determination of embryonic morphological landmarks and preservation of egg samples.” *International Journal of Legal Medicine* 130 (3): 845–54. https://doi.org/10.1007/s00414-015-1308-x.

Mellenthin, Katja, Khalid Fahmy, Reda A. Ali, Axel Hunding, Sol Da Rocha, and Stefan Baumgartner. 2006. “Wingless signaling in a large insect, the blowfly *Lucilia sericata*: A beautiful example of evolutionary Developmental Biology.” *Developmental Dynamics* 235 (2): 347–60. https://doi.org/10.1002/dvdy.20632.

Raminani, L.N., and E.W. Cupp. 1975. “Early embryology of *Aedes aegypti* (L.) (Diptera: Culicidae).” *International Journal of Insect Morphology and Embryology* 4 (6): 517–28. https://doi.org/10.1016/0020-7322(75)90028-8.

Ritter, Richard. 1890. “Die Entwicklung der Geschlechtsorgane und des Darmes bei *Chironomus." Zeitschrift Für Wiss Zool Bd* 50: 408–27.

Rohr, Klaus B., Diethard Tautz, and Klaus Sander. 1999. “Segmentation gene expression in the mothmidge *Clogmia albipunctata* (Diptera, Psychodidae) and other primitive Dipterans.” *Development Genes and Evolution* 209 (3): 145–54. https://doi.org/10.1007/s004270050238.

Rosay, Bettina. 1959. “Gross external morphology of embryos of *Culex tarsalis* Coquillett (Diptera: Culicidae).” *Annals of the Entomological Society of America* 52 (4): 481–84. https://doi.org/10.1093/aesa/52.4.481.

Sommer, R., and D. Tautz. 1991. “Segmentation gene expression in the housefly *Musca domestica*.” *Development* 113 (2): 419–30. https://doi.org/10.1242/dev.113.2.419.

Stefani, R N, D Selivon, and A L P Perondini. 2002. “Early developmental stages of *Ceratitis capitata* embryos.” *Proceedings of the 6th International Fruit Fly Symposium*, May: 55–58.

Strobl, Frederic, Marc F. Schetelig, and Ernst H.K. Stelzer. 2022. “In toto light sheet fluorescence microscopy live imaging datasets of *Ceratitis capitata* embryonic development.” *Scientific Data* 9 (1): 1–9. https://doi.org/10.1038/s41597-022-01443-x.

Suksuwan, Worramin, Xiaoli Cai, Lertluk Ngernsiri, and Stefan Baumgartner. 2017. “Segmentation gene expression patterns in *Bactrocera dorsalis* and related insects: Regulation and shape of blastoderm and larval cuticle.” *The International Journal of Developmental Biology* 61 (6–7): 439–50. https://doi.org/10.1387/ijdb.160277sb.

Telford, Allan D. 1957. “The pasture *Aedes* of Central and Northern California. The egg stage: Gross embryology and resistance to desiccation.” *Annals of the Entomological Society of America* 50 (6): 537–43. https://doi.org/10.1093/aesa/50.6.537.

Tollenaar, W., L. Joosten, and E. Schmitt. 2021. “The phases and effects of low temperature exposure on embryonic development in *Hermetia illucens* (L.) (Diptera: Stratiomyidae).” *Journal of Insects as Food and Feed* 8 (4): 379–86. https://doi.org/10.3920/JIFF2020.0165.

Turner, F.Rudolf, and Anthony P. Mahowald. 1977. “*Scanning electron microscopy of Drosophila melanogaster embryogenesis.*” *Developmental Biology* 57 (2): 403–16. https://doi.org/10.1016/0012-1606(77)90225-1.

Uliana, João Vitor Cardoso, Guilherme Thomaz Pereira Brancini, James Castelli-Gair Hombría, Luciano Antonio Digiampietri, Luiz Paulo Andrioli, and Nadia Monesi. 2018. "Characterizing the embryonic development of *B. hygida* (Diptera: Sciaridae) following enzymatic treatment to permeabilize the serosal cuticle." *Mechanisms of Development* 154: 270–76. https://doi.org/10.1016/j.mod.2018.08.002.

Urbansky, Silvia, Paula González Avalos, Maike Wosch, and Steffen Lemke. 2016. "Folded gastrulation and T48 drive the evolution of coordinated mesoderm internalization in flies." *eLife* 5 (September): 1–20. https://doi.org/10.7554/eLife.18318.

Vanario-Alonso, C. E., R. Sutton, J. C. Carvalho, M. Yussa, T. M. C. Silva, and E. Abdelhay. 1996. "Embryonic expression of the *engrailed* homologue of *Rhynchosciara americana.*" *Roux's Archives of Developmental Biology* 205 (7–8): 432–36. https://doi.org/10.1007/BF00377223.

Weismann, August. 1863. "Die Entwickelung der Dipteren im Ei nach Beobachtungen an *Chironomus spec.*, *Musca vomitoria* und *Pulex canis*." *Zeitschrift Für Wiss Zool Bd* 13: 107–220.

Wolf, Rainer. 1969. "Kinematik und Feinstruktur plasmatischer Faktorenbereiche des Eies von *Wachtliella persicariae* L. (Diptera)." *Wilhelm Roux' Archiv Für Entwicklungsmechanik Der Organismen* 162 (2): 121–60. https://doi.org/10.1007/BF00573537.

Wotton, Karl R., Eva Jiménez-Guri, Belén García Matheu, and Johannes Jaeger. 2014. "A staging scheme for the development of the scuttle fly *Megaselia abdita*." Edited by Peter K. Dearden. *PLoS ONE* 9 (1): e84421. https://doi.org/10.1371/journal.pone.0084421.

Wratten, Naomi S., Alistair P. McGregor, Philip J. Shaw, and Gabriel A. Dover. 2006. "Evolutionary and functional analysis of the tailless enhancer in *Musca domestica* and *Drosophila melanogaster.*" *Evolution & Development* 8 (1): 6–15. https://doi.org/10.1111/j.1525-142X.2006.05070.x.

Yoon, Yoseop, Jeff Klomp, Ines Martin-Martin, Frank Criscione, Eric Calvo, Jose Ribeiro, and Urs Schmidt-Ott. 2019. "Embryo polarity in moth flies and mosquitoes relies on distinct old genes with localized transcript isoforms." *eLife* 8 (October): 1–30. https://doi.org/10.7554/eLife.46711.

Supplementary Methods

Image processing and quantification

Surface projection. For *en face* views, the FIJI plugin Local Z Projector (Herbert *et al.* 2021 BMC Biol 19, 136. doi: https://doi.org/10.1186/s12915-021-01037-w) was used to project a surface of interest from a 3D stack onto a 2D surface, taking into account the curvature of the embryo. The reference plane that represents the contour of the embryo surface was derived from a Gaussian-blurred image of the original 3D stack image with a σ value of 2~4, followed by binarization with a customized threshold, which results in a smooth height map for z projection. For an optimal projection, three main parameters were considered, 1) median post filter for the height map, 2) ΔZ, and 3) offset for the maximum intensity projection. These parameters were standardized such that errors at the boundary of the embryo with the highest curvature and auto-fluorescence from the vitelline membrane closest to the topmost Z-slice were avoided. Whole embryo projections for embryos expressing Gap43-mCherry were done with ΔZ=0 and offset=3 or 4, while for embryos expressing 3xmScarletCaaX, ΔZ=0 and offset=2 or 3 were used. For embryos expressing MyoII-GFP and MyoII-mKate2, ΔZ=3 and offset=4 or 5 were used.

Re-slice along the z-axis. Re-slices were created using the re-slice tool in Fiji. A straight line approximately perpendicular to the CF or the head-trunk buckle was drawn and positioned between MD5 and MD9 to ensure the consistency of the slicing positioning along the D-V axis in all genotypes, except for *stg* mutants where there is no cell division and *khft* mutants where MD pattern is partially disrupted.

Time annotation. Time-lapse images of laterally mounted *D. melanogaster* embryos were aligned temporally based on the fact that the initiation timings of CF, VF and PMG are concurrent, marking the onset of gastrulation (Eritano *et al.* 2020 Developmental Cell 53, 2:212-240. doi:https://doi.org/10.1016/j.devcel.2020.02.012). For embryos imaged on the lateral side, the onset of PMG invagination was annotated based on a visual criteria of PMG surface flattening and the first major dorsal movement of the pole cells, with the exception of *khft* mutants in which the PMG was absent, and VF depth of approximately 60 µm was used to define gastrulation onset. For embryos imaged on the ventral side, the first frame at which the cells in the VF move out of plane or VF depth of approximately 60 µm was set as

gastrulation onset. Time-lapse images of laterally positioned *C. riparius* embryos were aligned temporally based on the observation that similar to *D. melanogaster* the initiation timings of VF and PMG are concurrent, marking the onset of gastrulation. For laterally imaged embryos (PMG not visible), the onset of gastrulation was determined based on a visual criteria of first collective ventral-ward cell movement. Extended Data Fig. 9a puts the dynamics of divisions in *D. melanogaster* and *C. riparius* head ectoderm on a common timeline based on these time annotations.

Measurement of CF or buckle depth. The depth of the CF or head-trunk buckling was measured at the time point where the invagination reaches the maximum depth. The segmented line tool in Fiji was used to trace the depth of the furrow starting from the vitelline membrane to the basal end of the epithelial cell at the tip of the CF or the head-trunk buckling.

Particle Image Velocimetry (PIV). PIV was performed on surface projections using the iterative PIV plugin in Fiji (https://sites.google.com/site/qingzongtseng/piv?authuser=0#h.khn09c6h1n39). Only the anterior half of the embryo was analyzed. Progressively decreasing Image interrogation window size of 88, 44 and 22 pixels was done with a search window of size 178, 88 and 44 respectively for each successive PIV iterations. The output of the 2nd iteration of PIV was used for the final vector plot. Time alignment of multiple embryos was based on gastrulation onset as described above. MD1 telophase onset was defined as one frame before the first MD1 cell entering cytokinesis. For the vector plot, vector coordinates were translated such that the x-axis origin was set at the CF or the head-trunk buckling, hence representing the head-trunk boundary, while the y-axis origin represents the lateral midline. For each time point (gastrulation onset and MD1 telophase), vector fields from three successive time frames were averaged. Averaged vectors from multiple embryos were plotted with the size of the arrow representing the magnitude of the velocity and, therefore, the flow speed. The vector lengths were extended by a factor of two for better visualization.

Nuclear density. Nuclear density was assessed at the end of cellularization by nuclear segmentation using 'Find Maxima' in FIJI, followed by manual corrections and Voronoi tessellation to generate a pseudo-cell territory around each maximum; territories at the edge of the image were excluded. The pseudo-cell territories were then dilated and merged to form

a contiguous area, in which the number of nuclei were counted to calculate the density. See also, Extended Data Fig. 8a–g.

Measurement of surface area in mitotic cells and regions. The surface area of individual mitotic cells or mitotically active regions was obtained by surface area segmentation of embryos expressing a fluorescent reporter for membranes using the TissueAnalyzer (Aigouy *et al.* 2016, Drosophila: Methods and Protocols Methods in Molecular Biology., pp. 227–239. https://doi.org/10.1007/978-1-4939-6371-3_13) plugin in FIJI. *C. riparius* embryos expressed Gap43-eGFP, and segmentation was performed on a single z-slice 4 µm below the cell apex; *D. melanogaster* embryos expressed 3xmScarlet-CaaX, and segmentation was performed on surface projections generated using the FIJI plugin Local Z Projector as described above. For the analysis of individual dividing cells, segmentation was carried out separately for each timepoint and cells were then tracked manually over time (24 out-of-plane and 18 in-plane dividing cells from 3 embryos). For each time point, the area of tracked cells was extracted. Following an in-plane division, both daughter cells were tracked and their area summed. Following an out-of-plane division, only the cell remaining at the surface was tracked; occasional short contacts of the bottom cell to the apical surface were not included in the analysis. Time annotation was performed respective to each individual cell's onset of telophase. For the quantification of mitotically active domains, the largest possible representative area of a given mitotic domain with fully traceable cells was chosen. In *C. riparius* embryos, domain areas were tracked and extracted manually for 5 embryos with time annotation based on the onset of gastrulation (described above). In *D. melanogaster,* domain areas were tracked and extracted manually for 6 embryos each. Due to slight differences in mitotic expansion timing relative to the onset of gastrulation between experiments and genetic backgrounds, maximal expansion of mitotic domains was used to normalize time. All cell and domain areas were normalized against their respective areas at the blastoderm stage.

Analysis of ventral midline deviation. For an estimate of deviation from the expected linear ventral midline, the ventral midlines visible on the ventral side were marked manually using the segmented line tool in FIJI at the onset of gastrulation (‘onset’) and a later time point (‘mid’) when the midline exhibits maximal distortion. The resultant ROIs were converted into points using the built-in macro function Roi.getContainedPoints in FIJI, and the coordinates were analyzed using a Python code. To compute deviation, a linear fit to the

midline coordinate was first generated as the expected midline for the onset stage, which was then translated to the linear fit for the midline coordinates marked at the 'mid' stage, while preserving the slope, as the expected midline for the mid stage. For each stage, the mean distance from the marked midline coordinates to the expected midline was calculated and plotted as 'Deviation' in Fig. 3c and Extended Data Fig. 7c.

Analysis of late-stage embryonic defects. Only datasets that showed normal CF formation (Sham-DNRho1) or successful optogenetic ablation of CF with subsequent buckling (Opto-DNRho1) were used for analysis. For both Sham-DNRho1 and Opto-DNRho1, embryos showing signs of widespread cell death during post-stimulation imaging were further excluded for analysis as these likely resulted from physical damage or phototoxicity. The remaining embryos were scored for three main morphological phenotypes: 1) tilts or twists in ventral midline (VM), 2) defects in head involution (HI), ranging from partial to complete failure, 3) defects in ventral nerve cord condensation (VNC), encompassing both incomplete condensation and commissure organization defects (disrupted or splayed). Subsequently, embryos were categorized based on the presence of a single or a combination of two or more phenotypes and plotted as a fraction of total embryos analyzed in the form of a pie chart (Fig. 4f and Extended Data Fig. 7f). Pairwise counting of phenotypes (HI+VNC, HI+VM or VM+VNC) was performed by calculating the percentage of embryos exhibiting both phenotypes in each pair, relative to the total number of embryos displaying at least one of the two phenotypes in that pair and plotted separately as bar graphs (Extended Data Fig. 7g).
